# A Clinical Text-Book of Surgical Diagnosis and Treatment for Practitioners and Students of Surgery and Medicine

**Published:** 1898-03

**Authors:** 


### Books and Magazines.

A CLINICAL TEXT-BOOK OF SURGICAL DIAGNOSIS AND TREATMENT FOR PRACTITIONERS AND STUDENTS OF SURGERY AND MEDICINE, by J. W. McDonald, M. D. Graduate in medicine of the University of Edinburgh; Licentiate of the Royal College of Surgeons, Edinburgh; Professor of the Practice of Surgery and of Clinical Surgery in Hamline University, Minneapolis, etc. With 328 illustrations. W. B. Saunders, Philadelphia, 1898. Price, cloth, $5; half morocco, $6 net.

The almost immeasurable advance in surgical science of the last few years has brought upon us almost an avalanche of surgical literature, surgical treatises in many volumes and almost of monthly occurrence, and the journals team with good, bad, and indifferent articles, and the mails are loaded with monographs. Truly we can paraphase the familiar hymn and say that "The half has never yet been read."

Tangled and hidden in this vast mass of debris, lie the most precious gems that human science has ever known. But after all the practical surgeon asks but two questions of his science when standing before his patient. First, what is the disease or injury? Second, what is the proper treatment?

In arriving at correct answers to these all-important questions, however, it is doubtless necessary for the surgeon to drink deeply from all sources of human learning. I have no patience with those narrow minds that would attempt to confine the medical man to the narrow limits of his own science. Much less then, should we attempt to exclude from our professional literature everything that is not intensely practical. But while admitting and advocating the necessity for an extensive literature in surgery, it is also of great advantage to the busy surgeon to have a convenient and compact statement of accepted practice for ready reference. The work under review comprises 798 pages, including a convenient index, and contains nothing but what is pertinent to diagnosis and treatment. The compass of the book, of course will not allow full discussion of all the bearings of these subjects, but is very valuable nevertheless as a book of ready reference, and any one who has not in his library a text-book less than three years old, should order this, as it is well known that accepted practice has greatly changed within this time. The numerous illustrations serve to greatly elucidate the text. An account of the origin and surgical application of the Roentgen rays is contained in the closing chapter.

